# NSAIDs-dependent adaption of the mitochondria-proteasome system in immortalized human cardiomyocytes

**DOI:** 10.1038/s41598-020-75394-x

**Published:** 2020-10-27

**Authors:** Laura Brandolini, Andrea Antonosante, Cristina Giorgio, Michela Bagnasco, Michele d’Angelo, Vanessa Castelli, Elisabetta Benedetti, Annamaria Cimini, Marcello Allegretti

**Affiliations:** 1 Dompé Farmaceutici SpA, Via Campo di Pile, L’Aquila, Italy; 2 Department of Life, Health and Environmental Sciences, University of L’Aquila, L’Aquila, Italy; 3 Sbarro Institute for Cancer Research and Molecular Medicine and Centre for Biotechnology, Temple University, Philadelphia, USA

**Keywords:** Biological techniques, Cell biology, Drug discovery, Cardiology

## Abstract

The progressive consumption growth of non-steroidal anti-inflammatory drugs (NSAIDs) has progressively raised the attention toward the gastrointestinal, renal, and cardiovascular toxicity. Increased risk of cardiovascular diseases was strictly associated with the usage of COX-2 selective NSAIDs. Other studies allowed to clarify that the cardiovascular risk is not limited to COX-2 selective but also extended to non-selective NSAIDs, such as Diclofenac and Ketoprofen. To date, although a less favorable cardiovascular risk profile for Diclofenac as compared to Ketoprofen is reported, the mechanisms through which NSAIDs cause adverse cardiovascular events are not entirely understood. The present study aimed to evaluate the effects of Ketoprofen in comparison with Diclofenac in immortalized human cardiomyocytes. The results obtained highlight the dose-dependent cardiotoxicity of Diclofenac compared to Ketoprofen. Despite both drugs induce the increase in ROS production, decrease of mitochondrial membrane potential, and proteasome activity modulation, only Diclofenac exposure shows a marked alteration of these intracellular parameters, leading to cell death. Noteworthy, Diclofenac decreases the proteasome 26S DC and this scenario may be dependent on the intracellular overload of oxidized proteins. The data support the hypothesis that immortalized human cardiomyocytes exposed to Ketoprofen are subjected to tolerable stress events, conversely Diclofenac exposition triggers cell death.

## Introduction

Non-steroidal anti-inflammatory drugs (NSAIDs) are widely used as prescribed, and in some cases over-the-counter (OTC), medications to alleviate inflammation, pain, and fever concomitant with various medical conditions^1^.

Whereas the use of NSAIDs is commonly associated with minor side effects, the progressive consumption growth has progressively raised the attention toward the gastrointestinal, renal, and cardiovascular toxicity^2^ profile of the class and numerous studies investigated the specific characteristics of each NSAID to better assess its risk/benefit profile^3^.

NSAIDs exert their pharmacological activity by inhibiting cyclooxygenases (COXs), a group of intracellular enzymes responsible for the conversion of arachidonic acid into prostanoids, biologically active lipids that finely regulate the inflammatory response^4,5^. Prostanoids include prostaglandins (PGs), prostacyclins (PGI2s), and thromboxanes (TXs), among which prostaglandins are key inflammatory mediators and clotting factors^4,5^. Three COX isoforms, COX-1, COX-2^6–8^, and COX-3^9^ have been described in humans, being the first two largely the most characterized and implicated in NSAIDs pharmacology.

COX-1, the constitutively expressed form of the enzyme, plays a pivotal role in many pathophysiological processes, such as platelet aggregation, gastric mucosa cytoprotection, and maintenance of renal function^6^.

While COX-2 although constitutively expressed in several human tissues including the central nervous system^10,11^, can be easily produced in response to pro-inflammatory cytokines or growth factors stimulation, thus being considered the most relevant mediator in promoting inflammation, fever, and pain^8^.

Increased risk of cardiovascular diseases emerged for the first time in the course of Vioxx Gastrointestinal Outcomes Research, or VIGOR, in 2000^12^ and, over the last decade, has been strictly associated with usage of COX-2 selective NSAIDs^13^. The original hypothesis was that the selective inhibition of COX-2 could result in a dramatic unbalance of the anti-thrombotic prostacyclin/pro-thrombotic thromboxane levels ratio thus favoring a pro-thrombotic environment potentially leading to clot formation and consequent cardiovascular damage^14,15^.

Further studies allowed to clarify that cardiovascular risk is not limited to COX-2 selective but is also extended to non-selective NSAIDs, commonly referred to as traditional NSAIDs (tNSAIDs), that may have at different degree adverse cardiovascular effect thus pointing towards the need for a deeper investigation on the underlying molecular COX-dependent and -independent mechanisms^14^.

Collected clinical evidence consistently points towards a less favorable cardiovascular risk profile for Diclofenac than naproxen, showing a similar risk between Diclofenac and other COX-2 inhibitors, at both high and low doses^16^.

To provide information on the risk/benefit profile of individual NSAIDs, the overall cardiovascular [acute myocardial infarction (AMI), heart failure (HF), acute ischemic stroke (IS)] and gastrointestinal risk evaluation was assessed within the SOS (safety of non-steroidal-anti-inflammatory drugs) project consortium, a multinational project funded by the European Commission. A wide, harmonized protocol was designed to conduct a nested case–control study based on electronic healthcare databases covering over 37 million people from four European countries: the Netherlands, Italy, Germany, and the UK. A statistically significantly higher risk of heart failure in association with the use of nine individual NSAIDs emerged. Among these NSAIDs indomethacin was found to increase the HF risk with an odd ratio (OR) 1.52, 95% confidence interval (CI) 1.31–1.77, and Diclofenac with an OR 1.21, 95% CI 1.16–1.26 whereas Ketoprofen was not associated with a significant risk of increased HF (OR 1.0, 95% CI 1.0–1.1), myocardial infarction (1.1) nor ischemic stroke (0.9). In 2012, it was published the final report of the SOS and the Group concluded that Ketoprofen, at any dose, does not present a risk of increased HF^17,18^.

Since the difference among individual NSAIDs is not fully explained based on the relative COX-2/COX-1 IC50 values, a large number of studies investigated the mechanisms through which NSAIDs cause adverse cardiovascular events showing that a complex network of targets and pathways is regulated by individual NSAID in the cardiovascular tissue^14,19^. These reports suggest that many NSAIDs may induce cardiomyocyte apoptosis with multiple mechanisms and that the increases in the rate of cardiomyocyte apoptosis could represent an essential step in the progression of HF^20–28^.

Oxidative stress caused by reactive oxygen species (ROS) generation may play a role in inducing apoptosis^29,30^. Many different NSAIDs have been shown to induce ROS formation. This action was observed in cultured gastric cells^31^ demonstrating that several NSAIDs, including Diclofenac, induce apoptosis by activating ROS production. NSAIDs susceptibility is more pronounced in heart tissue than other tissues of the body, as previously highlighted^32^. In a recent investigation, upon meclofenamate sodium and H_2_O_2_ exposure, the rat cardiac cells H9c2 and murine neonatal cardiomyocytes produced high ROS levels compared to kidney cells (CV1), skin fibroblasts and mouse embryo cells (CH3/10T/1/2)^33^.

Increased ROS levels were found to cause the opening of mitochondrial permeability transition pores (mPTPs) allowing the release of cytochrome c, the activation of caspase-9, and caspase-3, thus inducing apoptosis via the intrinsic/mitochondrial pathway^34^. Furthermore, the imbalance between physiological and pathological ROS levels could be associated with proteasome dysfunctions leading to decreased degradation of several proteasome substrates, including IκB, p53, Bax, and p27, and induced apoptosis^35,36^. ROS induction is not the only mechanism by which NSAIDs may induce proteasome dysfunction. Even though the mechanism is not fully understood, proteasome inhibition contributes to impair the proteasome protective function resulting in a higher risk of cardiac proteinopathy^37^.

A recent study demonstrated that, unlike aspirin, Diclofenac treatment in cardiomyocytes induced ROS generation, alterations of mitochondrial functions, and decreased proteasome activity^38^.

Hence, the present study aimed to evaluate the effects of Ketoprofen (K) in comparison with Diclofenac (Dic) in immortalized human cardiomyocytes.

## Results

### In vitro model characterization

As in vitro model, human immortalized cardiomyocytes were cultured as described in Materials and Methods. The characterization of the in vitro model was performed by assessing the localization of proteins specifically expressed in mature cardiomyocytes by immunofluorescence assay^39^.

**Figure 1:**
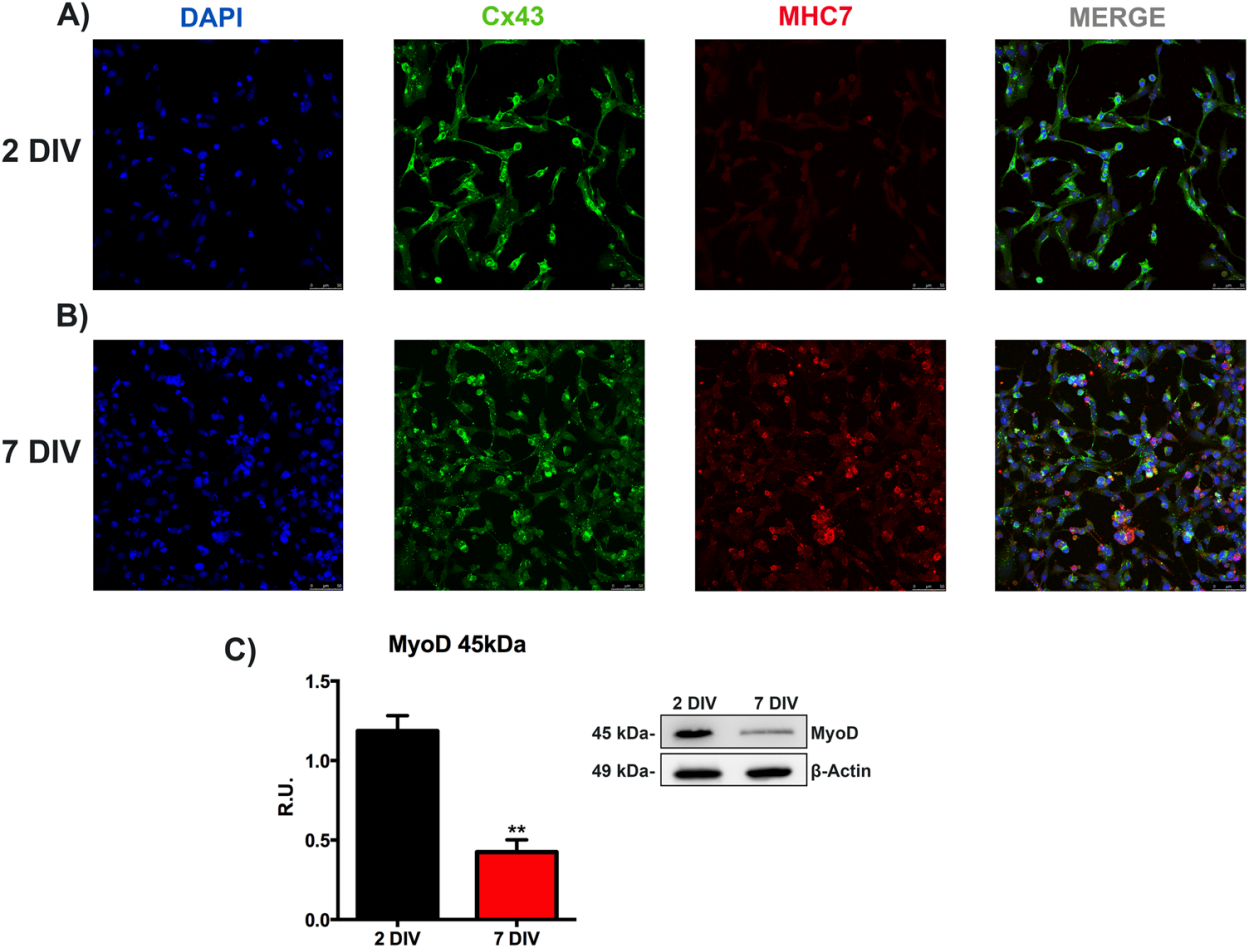
Analysis to characterize the in vitro model. Immunolocalization of proteins typically expressed in post-mitotic cardiomyocytes, at different times of culture. Confocal microscopy images are reported in (**A**,**B**). DAPI (blue), MHC7 (red), and Cx43 (green). For both the conditions, cells were grown in a medium supplemented with 10% of FBS for 24 h, then the cells were grown in 1% FBS-supplemented medium for one other day (**A**) (2DIV) and others 6 days (**B**) (7 DIV). Bar = 50 μm. (**C**) Western blotting analysis to evaluate MyoD protein levels. Data are mean ± SEM of three different experiments (*n* = 3). The image blot is a representative one. The cells were grown as described above. 2DIV versus 7DIV, **p < 0.01. Full-length blots can be found in Supplementary Fig. S1.

Confocal microscopy analyses were carried out by comparing the cells maintained for 24 h (48 h from the seeding, indicated as 2 days in vitro, 2DIV) in not-proliferating conditions with the cells maintained for six days in the same medium (seven days from the seeding, indicated as 7 days in vitro, 7DIV). The representative pictures of immunolocalization analysis of myosin heavy chain 7 (MHC7), a crucial late differentiation marker and connexin 43 (Cx43), the predominant gap junction in heart tissue, essential to ensuring cardiac electric activity^40^, are reported in Fig. 1A,B. Although we did not observe typical myosin striation, an increase in the red fluorescence intensity in 7DIV condition compared to 2DIV cells was observed. Cx43 immunostaining showed the increase of gap junction network in 7DIV cells compared to 2DIV cells. Myoblast determination protein 1 (MyoD), a skeletal muscle-specific bHLH transcription factor, which is early activated during myogenesis, and normally down-regulated in differentiated human cardiomyocytes^41,42^, was significantly reduced in 7DIV cardiomyocytes compared to 2DIV condition, as shown by western blotting analysis (Fig. 1C). These results provide evidence about the validity of this cellular model, as a useful tool to analyze the effects of NSAIDs on cardiac cells.

### Diclofenac affects cell viability in a dose-dependent way

**Figure 2:**
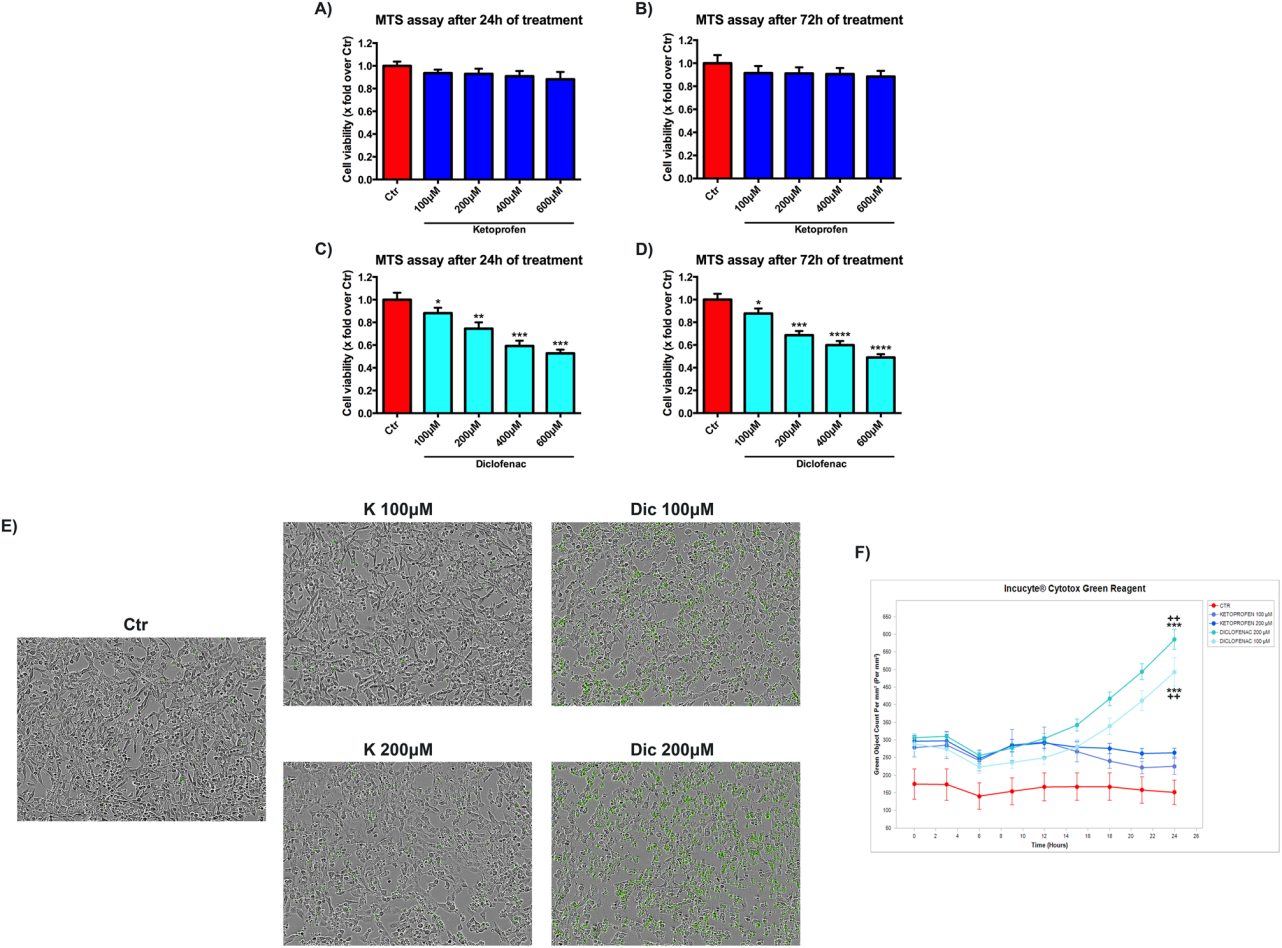
Diclofenac affects cell viability in a dose-dependent way. Cell viability assay (MTS) on immortalized human cardiomyocytes upon Ketoprofen (K) (**A**,**B**) and Diclofenac (Dic) (**C**,**D**) exposure for 24–72 h. Data are mean ± SEM of three different experiments run in quintuplicate; (*n* = 3). (**E**) Images of live-imaging Cytotox assay and F) real-time graph of green fluorescence quantification obtained by Incucyte device. Data are mean ± SEM of three different experiments run in six replicates; (*n* = 3). Ctr versus treated, *p < 0.05; **p < 0.01; ***p < 0.001, ****p < 0.0001; Keto 100 μM versus Diclofenac 100 μM, ^++^*p* < 0.01; Keto 200 μM versus Diclofenac 200 μM, ^++^*p* < 0.01.

The effects of the NSAIDs of interest on cell viability, in a concentration range of 100–600 μM for 24 and 72 h, were evaluated by MTS assay. K shows no toxic effects at any time and concentration considered (Fig. 2A,B). A significant dose-dependent reduction of cell viability was found upon Dic treatment without any correlation with time exposure (Fig. 2C,D). Based on these results, the concentrations of 100 and 200 μM of the NSAIDs of interest, were selected for all the subsequent experiments, to avoid high cellular mortality. Moreover, to get more insight in the decrease of cell viability observed in the MTS assay (that mainly measures the mitochondrial succinate dehydrogenase activity), live-imaging cytotoxicity assay (Incucyte Cytotox green) with both compounds was performed (Fig. 2E,F). The increase of green fluorescence, proportional to cytotoxicity, was displayed in cells exposed to both concentration of Dic compared to the untreated cells (Ctr), mainly after 24 h of treatment. Conversely, K-treated cells showed a slight increase of green fluorescence, at both the concentrations tested, compared to the untreated cells, thus indicating the cytotoxic effect of Dic only.

### Cell index analysis, Ketoprofen versus Diclofenac

**Figure 3:**
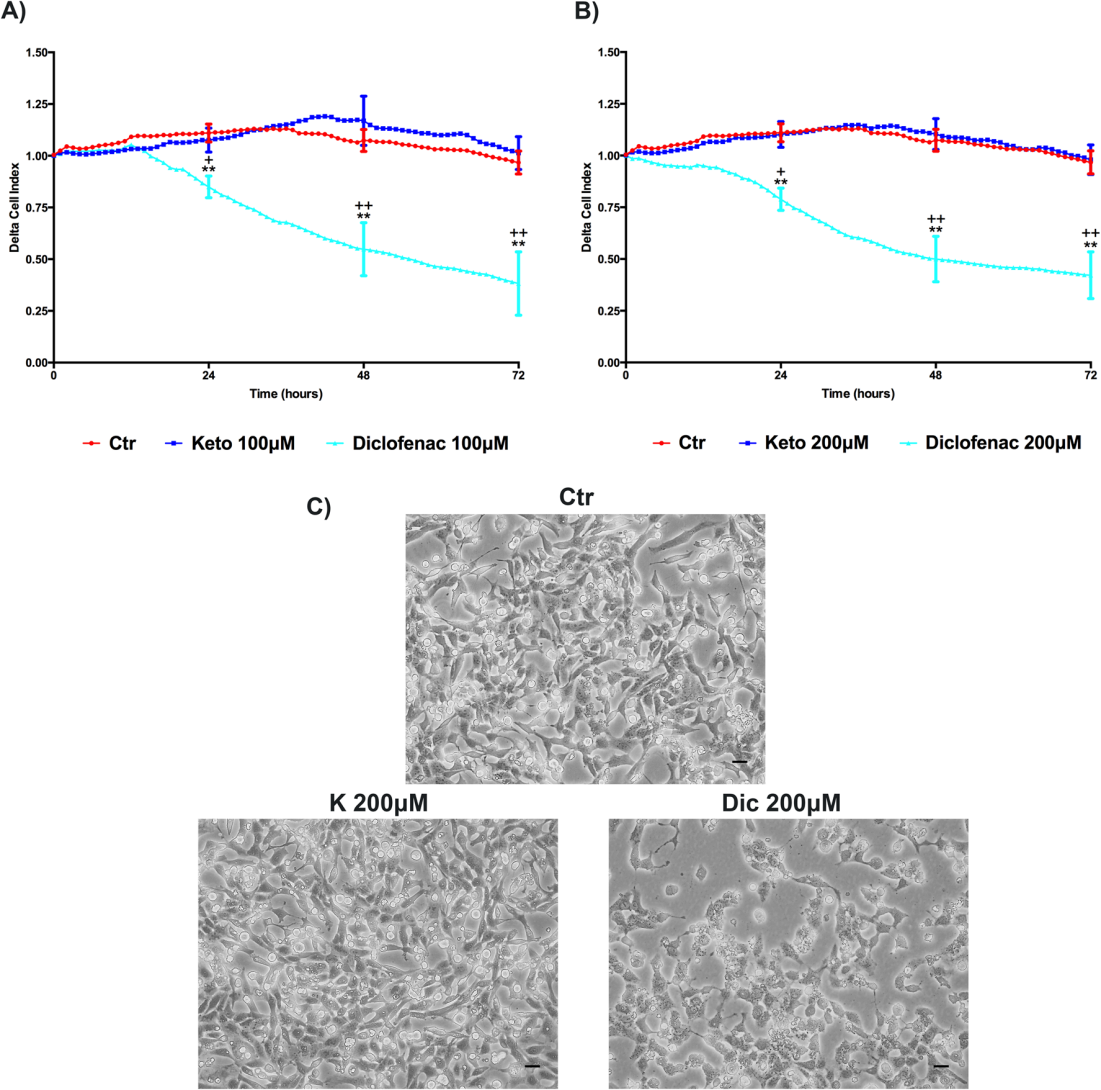
Delta Cell index (DCI) evaluation, K versus Dic, in immortalized human cardiomyocytes upon different treatment conditions. Cells were exposed to 100 μM (**A**) and 200 μM (**B**) of NSAIDs of interest. Data are mean ± SEM of seven different experiments run in triplicate; (*n* = 7). Ctr versus treated, **p < 0.01; Keto versus Diclofenac, ^+^*p* < 0.05, ^++^*p* < 0.01. (**C**) Contrast phase microscopy images of untreated and treated (for 24 h) not-proliferating immortalized human cardiomyocytes. Bar = 50 μm.

Cell Index (CI) was performed to provide a more sensitive and real-time analysis of cell health state compared to MTS, which only assays the cell metabolic activity. Delta cell index (DCI) related to 100 and 200 μM treatments are reported in Fig. 3A,B. K treated cells show a profile similar to the untreated cells. On the contrary Dic, at both concentrations, shows a significant reduction of DCI compared to untreated cells, as well as to K-treated cells. Furthermore, the cardiomyocytes morphological alterations due to Dic exposure, compared to K-treated and untreated cells, were also observed by contrast phase microscopy (Fig. 3C). It is possible to observe a morphological change upon Dic treatment.

### Different Ketoprofen- and Diclofenac-dependent effect on ROS imbalance

**Figure 4:**
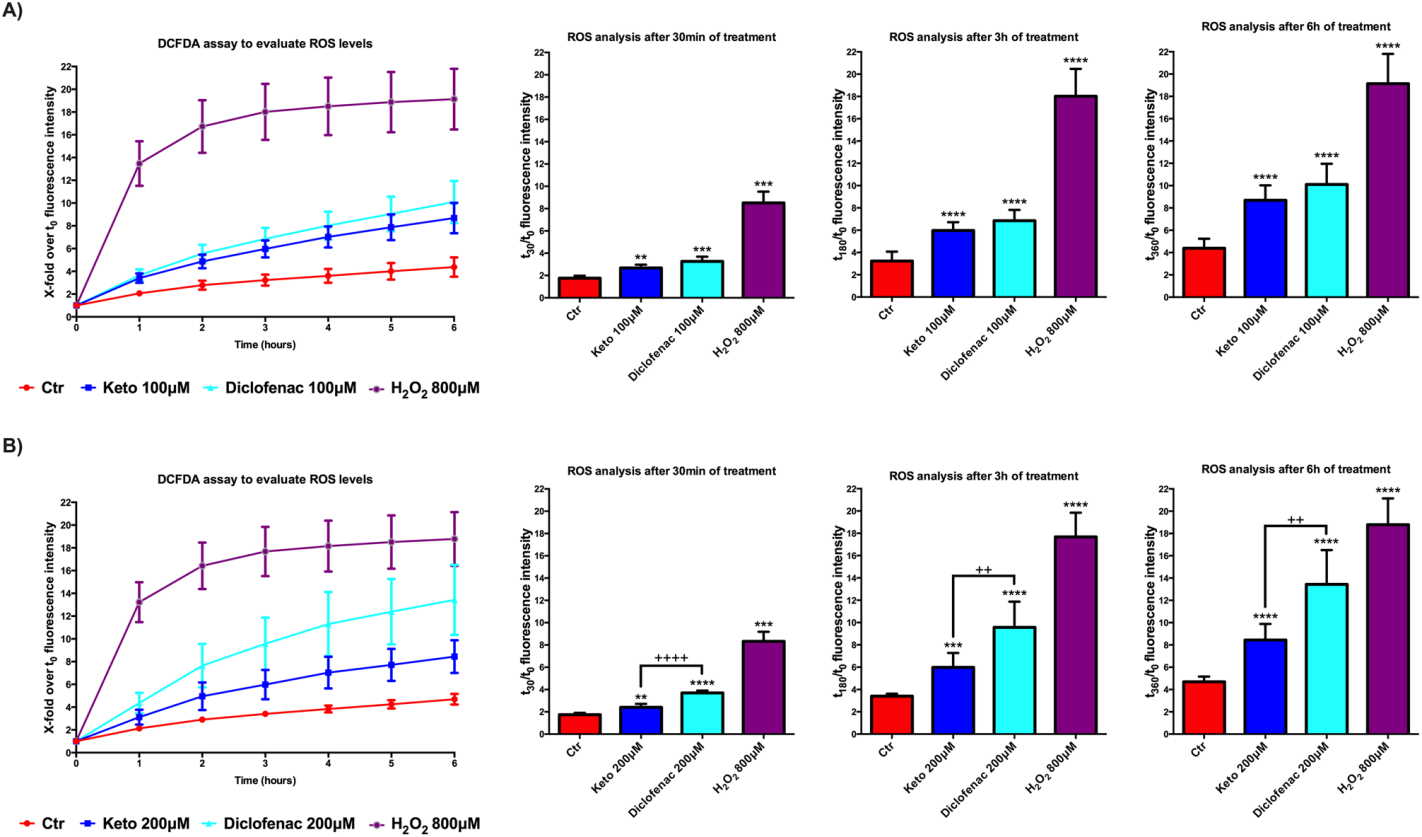
Different impact of K and Dic on ROS levels analysis in differentiated immortalized human cardiomyocytes. Cells upon K and Dic 100 μM (**A**) and 200 μM (**B**) exposure. Data are mean ± SEM of three different experiments run in quadruplicate; (*n* = 3). H_2_O_2_ 800 μM was used as Positive Control. The fluorescence intensity at each time point is indicated as the ratio of the value at a specific t-time point on the value at time point zero (first measurement) (t-time point/t0). Ctr versus treated, **p < 0.01, ***p < 0.001, ****p < 0.0001; Keto versus Diclofenac, ^++^*p* < 0.01, ^++++^*p* < 0.0001.

Most of NSAIDs, except the aspirin, are inducers of ROS imbalance toward pathological levels. Even NSAIDs without cytotoxic effects, such as naproxen sodium, were able to increase ROS levels, while Diclofenac and meclofenamate sodium showed increased ROS levels related to cytotoxicity^33,38^. On this basis, cardiomyocytes were treated with K and Dic 100, and 200 μM and ROS levels were measured by DCFDA assay (Fig. 4A,B). The assay was performed in the presence of the compounds during the first six hours of treatments; this is the optimal condition to measure ROS production, as suggested by the manufacturer. Already at 30 min from the starting of the assay, both compounds already show an upward trend of ROS levels compared to untreated cells. The differences between K and Dic are pronounced at the concentration of 200 μM, where Dic shows a significant increase of ROS levels compared to K (Fig. 4B).

### Ketoprofen and Diclofenac affect MMP in a different way

Mitochondrial metabolism is the primary ROS source; therefore the loss of normal mitochondrial homeostasis may result in the imbalance of ROS production. The electrochemical gradient between the inner and outer mitochondrial membranes drives the ATP synthesis and generates the mitochondrial membrane potential (MMP, Δ*Ψ*). MMP should be maintained in a homeostatic range to ensure the correct mitochondrial functions. This electrochemical parameter can be assayed to evaluate the mitochondrial state, which can be modulated by several xenobiotic compounds. In this regard, JC-1 cationic dye is an useful tool to detect MMP in adherent cells^43–45^. The reduction of aggregate/monomer ratio related to JC-1 dye indicates a decrease of the mitochondrial membrane potential resembling the effect induced by uncoupling agents, such as FCCP. This condition could be associated with cell death, but it is not mandatory, due to the possibility that some compounds induce a negative modulation of MMP without triggering cellular death pathways, as previously demonstrated by some investigations on salicylates^46–48^. Therefore, to evaluate the potential cytotoxicity of a specific compound, MMP detection assays should be supported by cell viability and cytotoxicity detection assays and by the assessment of mitochondrial number.

**Figure 5:**
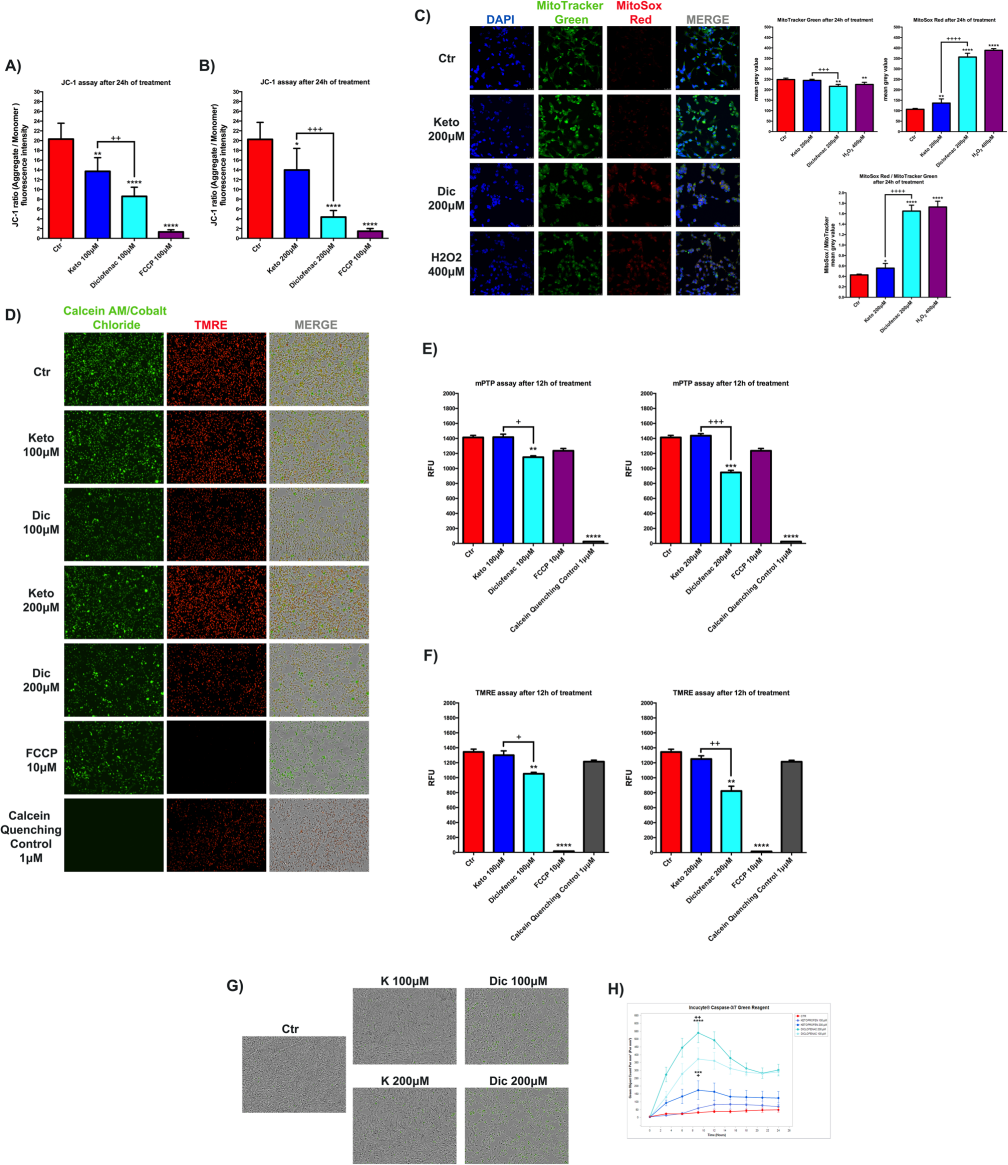
Ketoprofen and Diclofenac affect MMP differently. JC-1 assay to assess (MMP) in differentiated immortalized human cardiomyocytes upon K and Dic 100 μM (**A**) and 200 μM (**B**) exposure for 24 h. Data are mean ± SEM of four different experiments run in quadruplicate; (*n* = 4). FCCP 100 μM was used as Depolarization Control or uncoupling agent. The fluorescence intensity is indicated as the ratio between the fluorescence value of JC-1 aggregate form on the fluorescence value of JC-1 monomer form. (**C**) Images of MitoTracker Green and MitoSox Red staining and on the right, the histograms related to fluorescence intensity quantification expressed as mean grey value. DAPI was used to stain nuclei. H_2_O_2_ 400 μM for 1 h was used as MitoSox Positive Control. Data are mean ± SD of 5 fields/condition. Bar = 25 μm. (**D**) Images of live-imaging mPTP assay and (**E**) histograms of green fluorescence quantification (calcein-AM/cobalt chloride), (**F**) red fluorescence quantification (TMRE) obtained by Incucyte device. FCCP 10 μM for 12 h was used as Depolarization Control for TMRE, while calcein quenching control 1 μM was used as Negative Control for calcein-AM/cobalt chloride. Data were reported as RFU. (**G**) Images of live-imaging caspase-3/7 activation assay and (**H**) real-time graph of green fluorescence quantification obtained by Incucyte device. Data are mean ± SEM of three different experiments run in six replicates; (*n* = 3). Ctr versus treated, *p < 0.05, **p < 0.01, ***p < 0.001, ****p < 0.0001; Keto versus Diclofenac, ^+^*p* < 0.05, ^++^*p* < 0.01, ^+++^*p* < 0.001, ^++++^*p* < 0.0001. Keto 100 μM versus Diclofenac 100 μM, ^+^*p* < 0.05; Keto 200 μM versus Diclofenac 200 μM, ^++^*p* < 0.01.

We evaluated the MMP modulation induced by the NSAIDs of interest through the use of fluorescent cationic JC-1 dye and by TRME live imaging. A decrease of the JC-1 ratio at 100 (Fig. 5A) and 200 μM concentrations (Fig. 5B), after 24 h of treatments, is observed with both NSAIDs analyzed, Cells exposure to K and Dic at 100 μM (Fig. 5A) and 200 μM (Fig. 5B) for 24 h promoted a decrease of JC-1 ratio compared to the untreated cells, however, the effect of K was significantly less severe, especially at 200 μM, compared to Dic. This result is also supported by the TRME live analysis, showing a substantial reduction of red fluorescence intensity upon Dic only (Fig. 5F), while no decrease of TRME is observed with K. In line with previous results obtained by other research groups on cardiac tissue, cardiac cells^33,38^, hepatic tissue, and hepatocyte culture^49^, Dic exposure induces a detrimental reduction of MMP. On the other hand, it is possible to suppose that K effects on MMP result in a mechanism resembling the effect of salicylate derivatives on MMP, thus without significant cytotoxic events^46–48^.

All the observed events are, however, accompanied by a significant reduction of the mitochondrial marker Mitotraker upon Dic (Fig. 5C), thus suggesting a decrease of mitochondria in Dic-treated cells, not observed with K. Also, in Dic-treated cells a significant increase of the fluorescence intensity for MitoSox (a specific marker of mitochondrial superoxide) is observed (Fig. 5C), while this parameter is only slightly affected by K, thus suggesting a specific increase of mitochondrial superoxide in Dic-treated cells. Since the effect of ROS in triggering the opening of mitochondrial permeability pore (mPTP), inducing changes of mitochondrial membrane potential, likely activating intrinsic apoptotic pathway^34^; these parameters were assessed with live-imaging assay by Incucyte device. mPTP opening was evaluated by Mitochondrial PT Pore Assay. Briefly, this assays uses a calcein/cobalt quenching technique, where calcein stains the entire cell, while cobalt is able to quench the calcein fluorescence signal outside the mitochondrial matrix. If the inner mitochondrial membrane (IMM) is in physiological condition cobalt can not cross the IMM and the cells exhibit green fluorescence. Conversely, if the IMM is damaged the green fluorescence is quenched by cobalt, and the cells exhibit a decrease of the green fluorescence intensity.

Notably, upon Dic exposure (at both concentrations tested) the mPTP is significantly compromised compared to untreated and K-treated cells, as shown by the decrease of the green fluorescence intensity (Fig. 5D,E). These data were further supported by TMRE assay performed on the same cellular sample (Fig. 5D,F). Upon Dic exposure (at both concentrations tested) the red fluorescence intensity related to TMRE is decreased compared to untreated and K-treated cells.

Moreover, the induction of the apoptotic pathway was evaluated by Incucyte caspase-3/7 green assay, where the apoptosis activation appears proportional to the green fluorescence intensity.

In agreement with the previous data, after Dic exposure (at both concentrations tested), we observed a dramatic increase of caspase 3/7 activity, mainly after 10 h of treatment, compared to untreated and K-treated cardiomyocytes, as reported in Fig. 5G,H.

### Proteasome activity is modulated upon Ketoprofen and Diclofenac exposition

Altered intracellular levels of ROS may trigger protein damage resulting in proteotoxic stress and eventually in cell death^50^. In this scenario, the ubiquitin proteasome system (UPS) is deputed to recognition, degradation, and recovery (if possible), of damaged or unfolded proteins. The proteasome activities ensure the degradation of damaged proteins in an ATP-dependent process^51^. Proteasome or 26S proteasome is composed of three principal components, the core 20S or CP (core particle) containing proteolytic activity, and two ATP-dependent 19S or regulatory particles (RP). Three different protease activities within the proteasome core have been identified: caspase-like post acidic (β1), trypsin-like post basic (β2), and chymotrypsin-like post hydrophobic (β5). These core protease activities can be detected employing specific fluorescently tagged substrates. In intact substrate, the fluorescence is quenched, while after substrate cleavage the fluorescence is released and its intensity is proportional to proteasome activity therefore it can be detected. Despite the binding specificity of fluorescent compound used to detect proteasome activity, in the cellular crude extract, these fluorescent substrates can also be digested by other non-proteasome proteases. For this reason, the analysis should be performed in the presence and absence of a specific proteasome inhibitor, such as MG-132, bortezomib, or epoxomicin. To obtain a more reliable result from the assay, proteasome activity with the inhibitor is subtracted from proteasome activity without inhibitor^52–54^.

**Figure 6:**
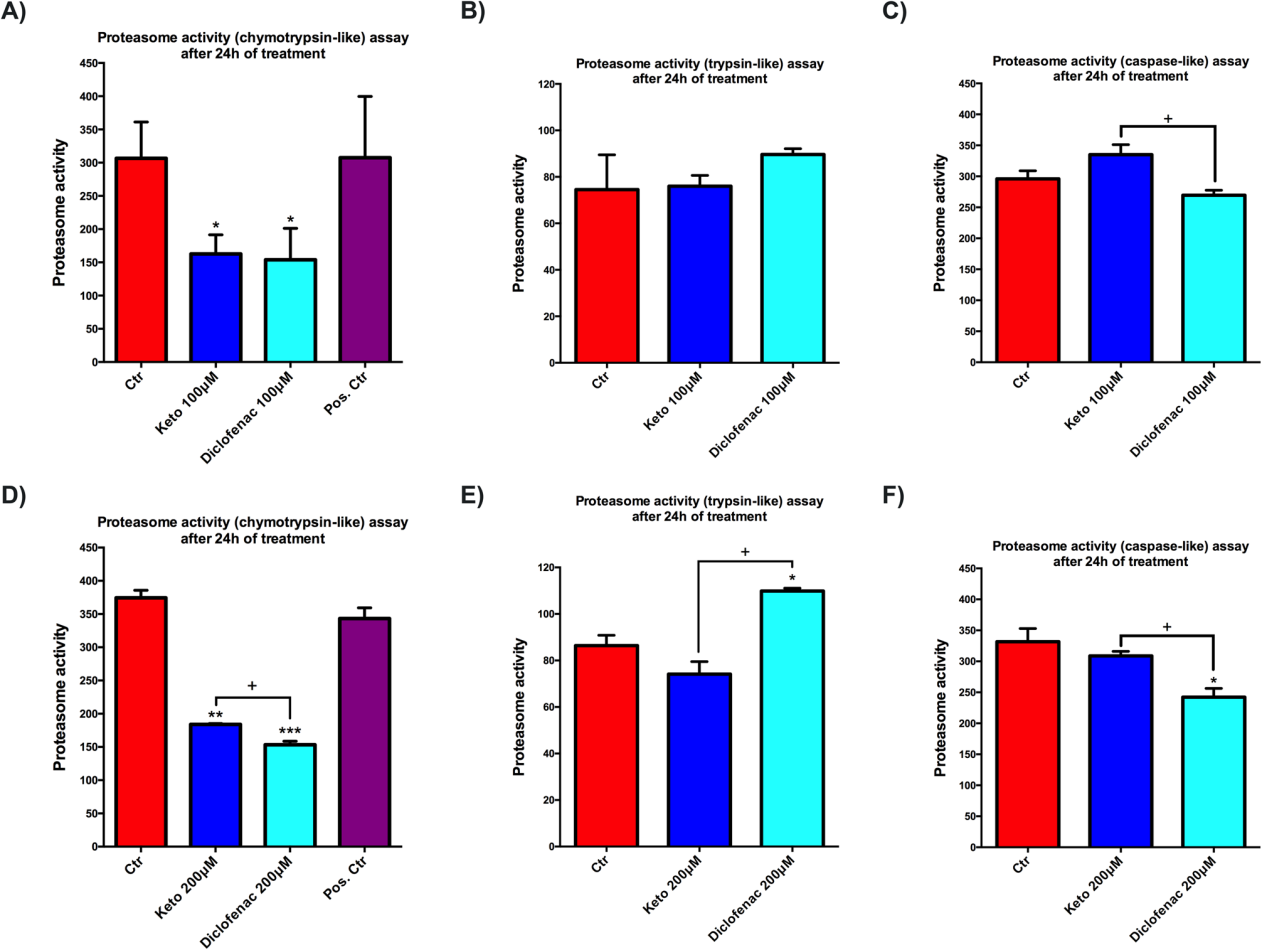
Proteasome activity is modulated upon Ketoprofen and Diclofenac exposition. Proteasome activity assay to evaluate (**A**,**D**) chymotrypsin-like (**B**,**E**) trypsin-like, and (**C**,**F**) caspase-like activity in immortalized human cardiomyocytes upon K and Dic 100 and 200 μM exposure for 24 h. Data are mean ± SEM of three different experiments run in triplicate; (*n* = 3). Jurkat cell lysate with significant proteasome activity was used as Positive Control. Trypsin-like and caspase-like activity lack positive control because it is provided only in the kit assay to evaluate chymotrypsin-like activity. Ctr versus treated, *p < 0.05, **p < 0.01, ***p < 0.001; Keto versus Diclofenac, ^+^*p* < 0.05.

We, therefore, analyzed the effects of tested NSAIDs, at 100 μM and 200 μM for 24 h, on chymotrypsin-like activity, the most recurring proteasome activity, in the presence and absence of MG-132 inhibitor (Fig. 6A,D). Both assessed compounds, at each concentration tested, show a significant decrease of chymotrypsin-like activity compared to untreated cardiomyocytes. Interestingly, a significant difference in this activity was observed only at high concentration in cells exposed to Dic compared to K (Fig. 6A,D). Although no significant differences between the compounds at 100 μM concentration are observed in trypsin-like activity (Fig. 6B), upon 200 μM concentration, cells treated with Dic show a significant increase of this proteasome activity compared to K-treated and untreated cells (Fig. 6E). Caspase-like activity is significantly reduced in cells upon Dic exposure compared to K-treated and untreated cells at both concentrations assayed (Fig. 6C,F).

### Diclofenac-dependent alteration of the proteasome configuration

**Figure 7:**
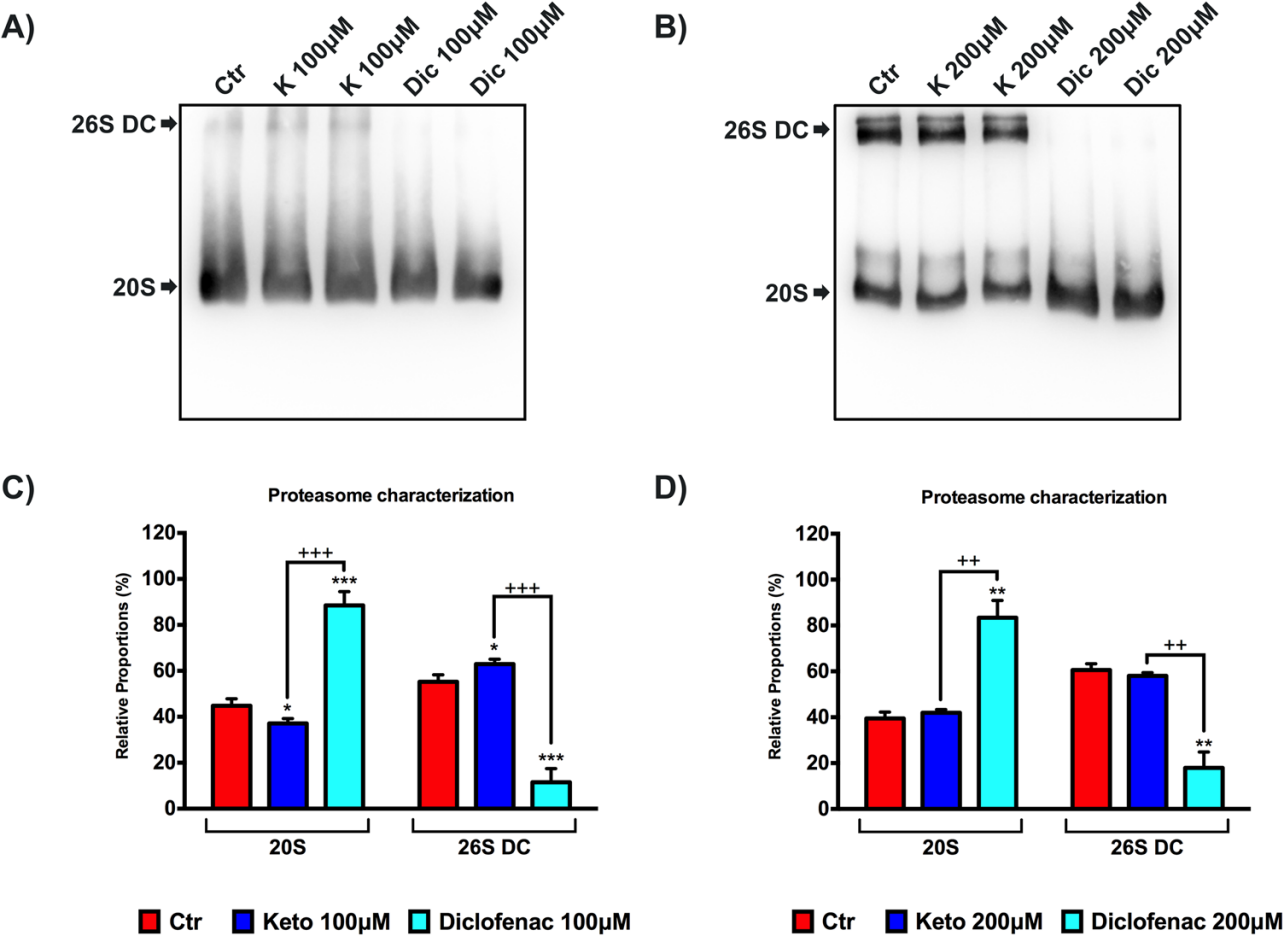
Diclofenac-dependent alteration of the proteasome configuration. Proteasome characterization by native gel electrophoresis/western in samples from differentiated immortalized human cardiomyocytes exposed to 100 and 200 μM of treatments for 24 h. (**A**,**B**) Western blotting representative membrane images. (**C**,**D**) Histogram of relative proportion (%) of the ratio between PSMA6 20S and PSMA6 26SDC, respectively, on tPSMA6 (total PSMA6 = PSMA6 20S + PSMA6 26SDC). Data are mean ± SEM of five different experiments; (*n* = 5). Ctr versus treated, *p < 0.05, **p < 0.01, ***p < 0.001; Keto versus Diclofenac, ^++^*p* < 0.01, ^+++^*p* < 0.001. Full-length blots can be found in Supplementary Fig. S2.

To support previous results, we assessed the effects of tested NSAIDs (100 μM and 200 μM for 24 h) on the proteasome structure by native gel electrophoresis/Western blotting, as described in previous investigations^33,38^. The representative images of the membranes are reported in Fig. 7A,B. The membranes were incubated with primary antibody anti-PSMA6 (proteasome sub-unit α6), which recognizes all proteasome structures. At both concentrations tested, in cells exposed to Dic, it is possible to observe a clear absence of the band corresponding to proteasome 26S double capped (26S DC); conversely, this band is present in untreated and K-treated cells (Fig. 7A,B). In Fig. 7C,D, the histograms of the relative proportion (in percentage) of each proteasome form (26DC and 20S) are reported. This is a qualitative analysis of proteasome configuration in a specific moment of cell life. The results show a proportional increase of proteasome 20S (about 80% of the total) and a clear proportional decrease of proteasome 26S DC (about 15% of the total) in immortalized human cardiomyocytes exposed to Dic. This effect is not found in cells exposed to K, which appear similar to untreated cells (Fig. 7C,D). Hence, we can postulate that Dic induces alterations of 26S structure, which establishes a qualitative loss of proteasome configuration.

### Increased levels of intracellular oxidized proteins after Diclofenac exposition

**Figure 8:**
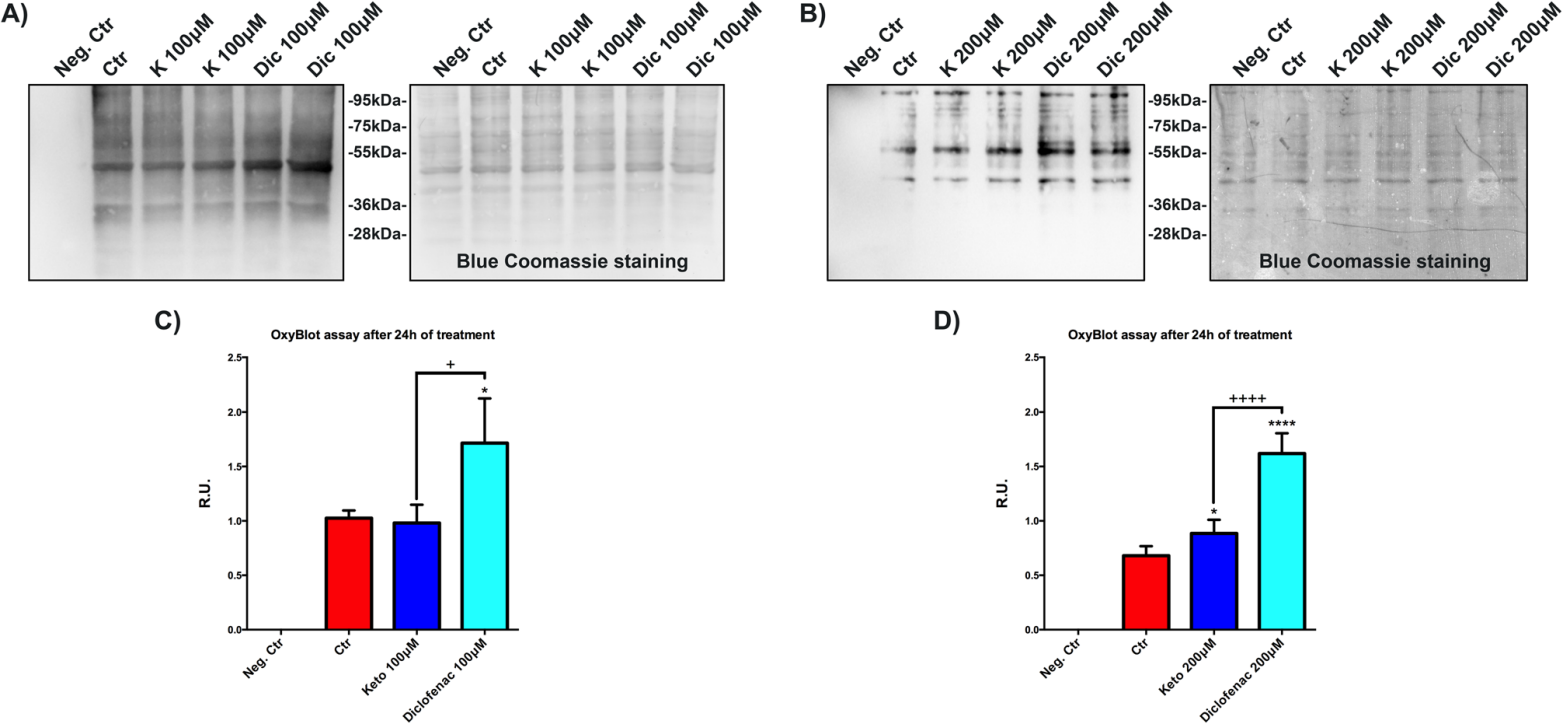
Increased levels of the intracellular oxidized proteins after Diclofenac exposition. OxyBlot assay to assess the oxidized protein levels. (**A**,**B**) Oxyblot representative image with the respective image of the membrane with blue coomassie staining. (**C**,**D**) Histograms of OxyBlot densitometric analysis. Data are mean ± SEM of three different experiments; (*n* = 3). Data were expressed as R.U., and each sample was normalized on its respective blue coomassie staining. Neg. Ctr (negative control) is a sample without protein derivatization. Ctr versus treated, *p < 0.05, ****p < 0.0001; Keto versus Diclofenac, ^+^*p* < 0.05, ^++++^*p* < 0.0001. Full-length Oxyblot and membranes stained with blue coomassie can be found in Supplementary Fig. S3.

High levels of oxidized proteins, due to non-physiological oxidative stress levels associated with mitochondrial damage, induce the proteasome 26S dismantling^55–58^. In this situation the UPS is ineffective, and only proteasome 20S can remove oxidized proteins. Oxyblot assay is used to evaluate the intracellular levels of oxidized proteins. In Fig. 8A,B representative images of OxyBlot with respective blue Coomassie staining of the same membrane are reported. In Fig. 8C,D, the histograms of the respective analyses are reported. In our experimental conditions, the immortalized human cardiomyocytes exposed to Dic show a marked increase of oxidized protein compared to untreated cells and K-treated cells, suggesting an accumulation of oxidized proteins that may be involved in the replacement of 26S with 20S proteasome in cardiomyocytes, as previously demonstrated^55–58^.

## Discussion

Reports on cardiovascular adverse reactions began to emerge in early 2003^59^. Later, several placebo-controlled trials focused on COX-2 inhibitors showing an increased risk of atherothrombotic vascular events associated with the use of these drugs^60,61^. More recent data from meta-analyses of randomized trials and observational studies have contributed to clarifying that cardiovascular side effects are not a peculiar characteristic of COX-2 inhibitors, but are also associated with the use of some NSAIDs^60–67^.

Several findings support the concept that both COX-2 selective inhibitors and tNSAIDs may increase cardiovascular risk, although this effect greatly varies among individual drugs and strictly depends on the dose^62^. The difference among individual NSAIDs-associated side effects requires specific investigation since it may depend in part on the specific pharmacodynamic properties, but it is very likely to rely also on unique COX-independent activities of the molecule.

As previously illustrated, COX-2 selective inhibition by some NSAIDs, such as celecoxib and Diclofenac, was considered the main cause of the increased cardiovascular risk since the imbalance between thrombogenic (thromboxane) and anti-thrombogenic (prostaglandins) factors due to COX-2 inhibition may favor thrombotic events which can trigger and exacerbate cardiovascular disease^16,68^.

Increased levels of 20-Hydroxyeicosatetraenoic acid (20-HETE) were observed in mice treated with COX-2 inhibitors and associated with decreased tail bleeding and increased platelet aggregability^69^. Although increased 20-HETE levels are likely related to COX-2 inhibition and probably contribute to the adverse cardiovascular outcome, this effect was observed across the NSAIDs class thus excluding a specific COX-2 dependent effect.

Many recent reports suggest that NSAID-induced increase in the rate of cardiomyocyte apoptosis has a significant effect on heart function and is implicated in the progression of HF^20–28^.

Prostaglandins activate a class of receptors called E-type prostanoid (EP) receptors, which play a key role in the development of pain and inflammation and are also involved in the control of apoptosis and cell survival^70,71^.

Thus, the block of prostaglandin signaling by NSAIDs may be responsible for NSAID-induced apoptosis. However, other mechanisms are emerging that better explain the individual behavior of different NSAIDs. Specifically, NSAID-induced ROS generation in cardiomyocytes was found to be a critical step in the induction of apoptosis, associated with mPTPs opening and proteasome dysfunction^32–38^.

NSAID-induced ROS can damage proteins causing multiple effects on the proteasome: the oxidation of proteasomal subunits may indirectly result in proteasome inhibition but the increased levels of oxidized proteins may also overload the proteasome, inducing dysfunction.

To better understand the COX-2 independent effects accounting for cardiovascular side effects, we compared the effects of Ketoprofen and Diclofenac in differentiated immortalized human cardiomyocytes.

Interestingly, the results obtained in the in vitro model revealed a markedly deleterious effect of Dic in comparison to K. We show that Dic exposure causes cardiotoxicity and a strong decrease of cell viability, in line with a recent investigation^38^. Considering the cardiomyocytes attitude to produce ROS during the metabolic activity to ensure the lifespan of cardiac tissue, it is conceivable to assume that a correct balance between physiological and pathological ROS levels is indispensable for cellular homeostasis. Some NSAIDs, such as naproxen sodium, are ROS inductors without cytotoxic effect, while others, such as Diclofenac and meclofenamate sodium, are strong ROS inductors associated with cytotoxicity^33,38^.

Mitochondria play a crucial role in cellular ROS production, therefore the loss of normal mitochondrial homeostasis results in an imbalance of ROS generation. Some NSAIDs can uncouple oxidative phosphorylation and dissipate MMP as extensively demonstrated^72^. In our experimental conditions, K treatments did not affect cell viability, while increases total ROS production, but not mitochondrial ROS and slightly decreases MMP in a dose-independent way, without affecting the mitochondrial number. On the contrary, Dic treatments strongly altered these parameters in a dose-dependent manner, reducing mitochondrial number and increasing mitochondrial ROS, leading to cell death. In summary, a potential tolerable effect of K on ROS production and MMP was observed, in agreement with that described for salicylate derivatives and naproxen^33,47,48^.

Conversely, in our experimental conditions, after Dic administration, higher ROS production (both total and mitochondrial) associated with a strong mitochondrial membrane depolarization and a decrease of mitochondria was found. Although K exposition triggers a total ROS imbalance, it appears not detrimental and may induce, as a consequence, the expression of cytoprotective genes, as already demonstrated^73^, resembling the cellular response to physiological levels of ROS. Conversely, Dic triggers a marked ROS imbalance (both total and mitochondrial), which is proportionally linked to the exposure dose. Due to high ROS-induced stress, Dic-treated cells are unable to trigger cytoprotective response, thus favoring cell deathlikely by apoptosis as demonstrated by activation of caspases 3/7.

The underlying mechanism of NSAIDs-dependent depolarization effect may be due to their ability toactivate the mPTPs opening, which ensures the free passage of low molecular weight compounds between the inner mitochondrial matrix and cytosol. The opening of mPTPs is promoted by Ca^2+^ accumulation in mitochondria, pro-oxidants, and low MMP. In this regard, NSAIDs such as aspirin and derivatives, directly affect mPTPs resulting in depolarization of the mitochondrial membrane, as previously described^46–48^. This action is linked to impaired mitochondrial Ca^2+^ uptake, as already observed in colon cancer cell lines^48^ and vascular smooth muscle cells (VSMCs)^47^. The proton conductance of the inner mitochondrial membrane is increased by salicylate, thus this net proton influx leads to the uncoupling of mitochondria^46^. Our hypothesis on the observed K-dependent mitochondrial membrane depolarization is that the drug has the ability to affect proton influx in the mitochondrial respiratory chain, similarly to salicylate derivatives. Also, it was previously shown that the uncoupling effect of Dic is about 50-fold greater than salicylate^74^. In agreement, our results show an alteration of mPTP in cells exposed to Dic; conversely cells exposed to K show the same behaviour of untreated cells. These data were further confirmed by TMRE staining, where cells exposed to Dic show a significant decrease in the fluorescence related to TMRE. Therefore, Dic enhances the permeability of transition pore on the IMM, which, in turn, may induce the depolarization of mitochondrial membrane leading to activation of the apoptotic pathway.

As mentioned above, mitochondria metabolism plays a central role in ROS production. Non-physiological levels of ROS, together with inadequate antioxidant defences trigger protein damage that results in proteotoxic stress and eventually in cell death^50^. The management of damaged or unfolded proteins is mediated by UPS, which delivers ubiquitin tagged proteins to the proteasome and ensures their degradation. UPS is an ATP-dependent process and each event is realized with energy consumption to counteract intra- and extra-cellular proteotoxicity^51^. The proper activity of UPS is responsible for the correct turnover of proteins that are required for cardiac homeostasis. Impaired UPS function has been implicated in heart diseases^75,76^.

Proteasome or 26S proteasome is composed of three principal components, the core 20S or CP with proteolytic activity, and two ATP-dependent 19S or RP. Four stacked rings with a central cavity form the core particle. These rings are arranged in a particular manner: two outer rings composed of seven α-subunits and two inner rings composed of seven β-subunits. Only three β-subunit present the active protease site, each one diverging from the others due to the different amino acidic cutting site recognized. To date, three different protease activities within the proteasome core have been identified: caspase-like post acidic (β1), trypsin-like post basic (β2), and chymotrypsin-like post hydrophobic (β5). The regulatory particle is composed of ATPase subunits accountable for protein cargo translocations in the 20S core. Subunits composing 19S particles can bind ubiquitin, thus participating in the recognition and de-ubiquitination of substrates^51,77^. Each of the three 20S core protease activities can be detected utilizing specific fluorescently tagged substrates. Our results show the direct role of Dic in triggering 26S structure alterations, which establish the qualitative loss of proteasome activity. Although 26S proteasome is involved in the recognition and degradation of ubiquitinated proteins, the loss of 19S triggers an alternative mechanism of degrading damaged proteins, which is dependent only on 20S proteasome activity. Therefore, sustained stress conditions into the cell, such as excessive production of ROS, could reduce UPS activity leading to disassembling of proteasome 26S. When two 19S regulatory particles are separated from the 20S core, the degradation of damaged proteins is carried out only by 20S core, without ubiquitin labeling and ATP consumption. This condition is especially induced by high oxidative stress^55–58^.

Although proteasome chymotrypsin-like activity is negatively affected by K exposure, it is possible to hypothesize that cells treated with K maintain a functional UPS, like untreated cells, since they shows an intact 26S proteasome, also called 26S DC. As a matter of fact, in our experimental condition, upon K exposure the immortalized human cardiomyocytes preserve proteasome caspase-like activity. On the contrary, cells treated with Dic show only 20S proteasome structure and a significant loss of chymotrypsin-like and caspase-like proteasome activity. Noteworthy, upon Dic exposure cardiomyocytes show a significant increase of trypsin-like proteasome activity. Cytotoxic events due to Dic exposure are probably associated with excessive ROS production, leading to a marked increase of oxidized proteins.

In this context, ATP production could be limited, and oxidized protein levels may increase. The concurrence of these events could trigger the disassembly of proteasome 26S and the loss of ubiquitin-dependent proteasome activity. The high amount of damaged protein could accumulate into the 20S core and then overload proteases activity leading to a reduction in the overall proteasome activity, as observed after Dic exposure.

**Figure 9:**
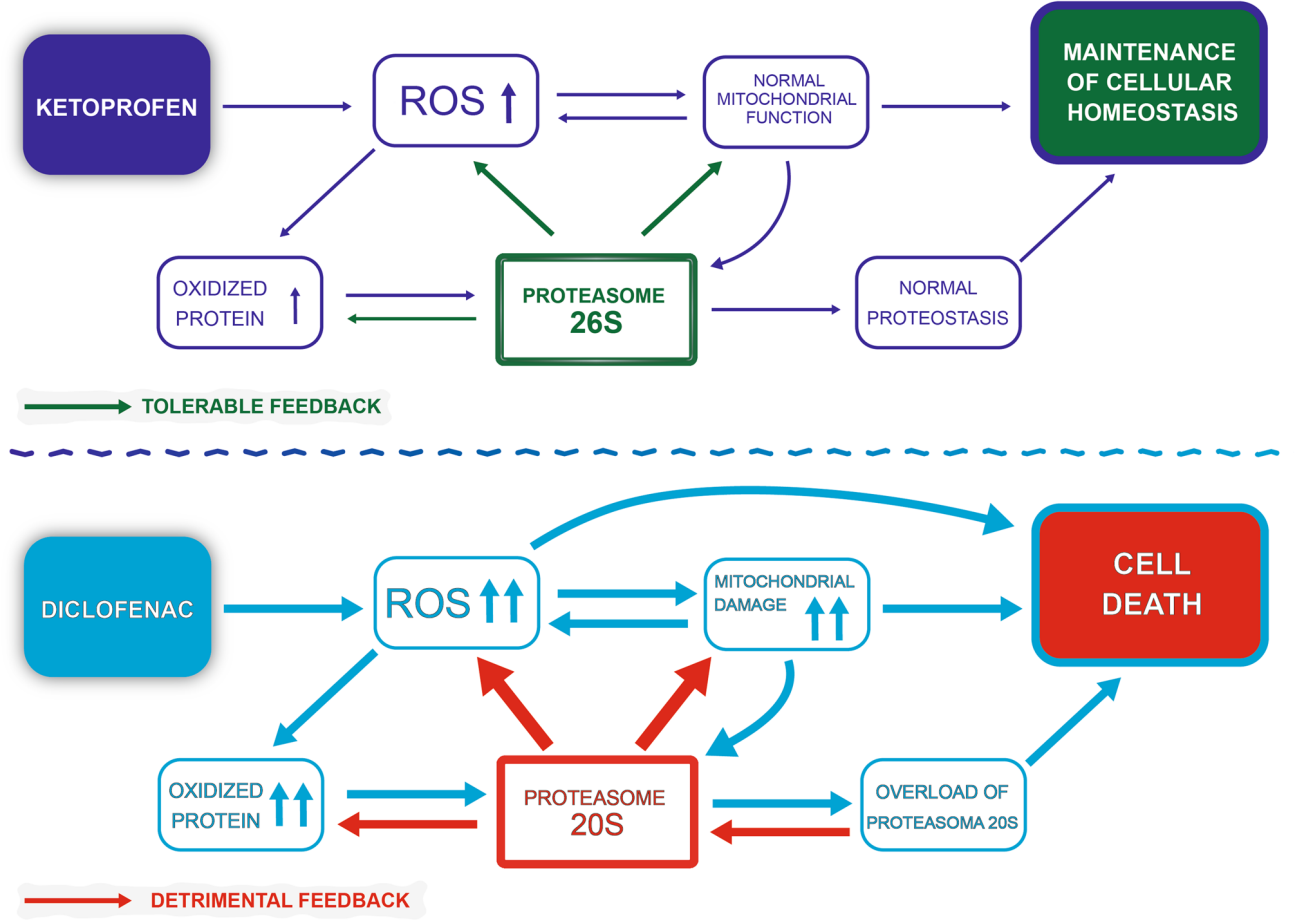
Schematic representation of the proposed mechanism of K versus Dic.

Probably, immortalized human cardiomyocytes exposed to K maintain responsiveness to counteract tolerable levels of stress, conversely Dic treatment triggers a cardiotoxic response, ultimately leading to cell death as summarized in Fig. 9, where a possible sequence of events is depicted for both compounds. .

## Materials and methods

### Immortalized human cardiomyocytes culture

Immortalized human cardiomyocytes cells were used as a cardiac model and purchased from Applied Biological Materials Inc. (abm). They derived from the ventricular tissue of 62 old years male. The culture media was composed of Dulbecco’s modified Eagle’s medium/Ham’s F12 50/50 mix containing 10% Foetal bovine serum (FBS), 100 U/ml penicillin, 100 U/ml streptomycin, 2 mM glutamine (Corning, Manassas, VA, USA) and it was replaced day by day. The cells (used at passage 4–8) were subcultured by 0.25% trypsin–EDTA (Corning, Manassas, VA, USA) enzymatic digestion. For cardiomyocytes characterization, cells were seeded in 10% FBS supplemented DMEM F12 at a seeding density of 2.5 × 10^4^ cells/cm^2^. 24 h after seeding, 10% FBS supplemented DMEM F12 was replaced with the culture medium supplemented with 1% of FBS, which was changed every day until the sixth day. Then, human cardiomyocytes were exposed to NSAIDs of interest.

### Cell treatments

Ketoprofen powder was dissolved in sterile water (containing 20 μl of NaOH 5 N) to obtain a 50 mM stock solution, and used in a final concentration range of 100–600 μM. Diclofenac powder was dissolved in sterile DMSO (Sigma, St. Louis, Mo, USA) at an initial concentration of 50 mM and used in a final concentration range of 100–600 μM. Each solution was freshly prepared for every experiment. As mentioned above, seven days after seeding, (seeding density of 2.5 × 10^4^ cells/cm^2^, one day in 10% FBS-supplemented medium and 6 days in 1% FBS-supplemented medium) cardiomyocytes were exposed to each treatment for 24–72 h.

### Contrast phase images

Immortalized human cardiomyocytes were seeded into a collagen I-coated flask T75 cm^2^ (2.5 × 10^4^ cells/cm^2^ seeding density) in 10% FBS supplemented DMEM F12. 24 h later the 10% FBS supplemented media was replaced with 1% FBS supplemented media, and the cardiomyocytes were maintained in this culture conditions for 6 days. On the seventh day of culture, the cells were exposed, for 24 h, to 200 μM of NSAIDs in 1% FBS-supplemented medium. Then the images were acquired at 20 × magnification by Leica DMi1-CH-9435 optical microscope.

### Immunofluorescence

Immortalized human cardiomyocytes were seeded on collagen I-coated (10 μg/ml) (Sigma, St. Louis, Mo, USA) sterile glass coverslips (2.5 × 10^4^ cells/cm^2^ seeding density) in 10% FBS supplemented DMEM F12. 24 h later the 10% FBS supplemented media was replaced with 1% FBS supplemented media, and the cardiomyocytes were maintained in this culture conditions for 24 h and six days, to perform the analysis for cellular model characterization. Cells were fixed in 4% formalin (Sigma, St. Louis, Mo, USA) for 10 min at room temperature (RT) and then rinsed thrice with phosphate-buffered saline (PBS) (Corning, Manassas, VA, USA). After permeabilization (Triton X-100 (Sigma, St. Louis, Mo, USA) 0.1% in PBS for 5 min at RT) and three washes, the cells were incubated for 20 min in PBS containing 4% bovine serum albumin (BSA) (Sigma, St. Louis, Mo, USA) at RT, and successively incubated overnight at 4 °C with the primary antibodies. Subsequently the cells were rinsed four times with PBS, and incubation for 1 h at RT with the secondary antibodies was performed. Primary antibodies were mouse anti-myosin heavy chain 7 (MHC7) (1:200) and rabbit anti-connexin 43 (Cx43) (1:400) purchased from Abcam (Cambridge, UK) (Invitrogen, Life Technologies, Foster City, CA, USA). Alexa Fluor-488 goat anti-rabbit (1:2000) Alexa Fluor-633 and anti-mouse (1:2000) (Invitrogen, Life Technologies, Foster City, CA, USA) were used as secondary antibodies. Finally, coverslips were mounted on microscope slides with Vectashield Mounting Medium with DAPI (Vector Laboratories Inc, Burlingame, CA, USA) and observed with Leica TCS SP5 confocal microscope (Mannheim, Germany).

### MitoTracker Green and MitoSox Red staining

Immortalized human cardiomyocytes were seeded on collagen I-coated [10 μg/ml] (Sigma, St. Louis, Mo, USA) sterile glass coverslips (2.5 × 10^4^ cells/cm^2^ seeding density) in 10% FBS supplemented DMEM F12. 24 h later the 10% FBS supplemented media was replaced with 1% FBS supplemented media, and the cardiomyocytes were maintained in this culture conditions for six days. On the seventh day of culture, the cells were exposed, for 24 h, to 200 μM of NSAIDs in 1% FBS-supplemented medium. H_2_O_2_ 400 μM for 1 h was used as MitoSox positive control. MitoTracker Green and MitoSox Red (both from Invitrogen, Life Technologies, Foster City, CA, USA) were used according to manufacturer’s instructions. Live cells were exposed to 200 nM of MitoTracker Green for 15 min in an incubator, while MitoSox Red final concentration used was 5 μM for 10 min in the incubator. Finally, coverslips were mounted on microscope slides with Vectashield Mounting Medium with DAPI (Vector Laboratories Inc, Burlingame, CA, USA) and observed with Leica TCS SP5 confocal microscope (Mannheim, Germany). For fluorescence quantification digital images (5 fields/condition) were analyzed by Image J Software, the signal intensity (in arbitrary units) was provided as mean grey value.

### Cell viability evaluation

Immortalized human cardiomyocytes were treated (seeding density 2.5 × 10^4^ cells/cm^2^) after seven days from the seeding. The treatment exposition was 24 and 72 h at 100–600 μM. CellTiter 96 AQueous One Solution kit MTS test assay (Promega, Madison, WI, USA) was used to assess cell viability. Absorbance related to living cells was measured at 492 nm by a spectrophotometric microplate reader (Infinite F200, Tecan, Männedorf, Switzerland). The absorbance ratio of the treated cells/untreated cells was used to express the results.

### Cell index (CI)

Immortalized human cardiomyocytes were seeded into the wells of 16-well E-plate at a seeding density of 2.5 × 10^4^ cells/cm^2^ and cultured for seven days. Each treatment was performed after the seventh day of culture; a suitable time to obtain differentiated human cardiomyocytes.

The xCELLigence system (Roche Applied Science) provides a quantitative parameter called cell index (CI), which reflect the cell status. Briefly this system measures cell-electrode impedance, thus the CI represents a quantitative measure of the cell number, cell viability, adhesion degree, and morphology. The results are reported as delta cell index (DCI). For each well, DCI represents the CI at a given time point (CI_ti_) plus delta value. The difference between a reference DCI value and the CI at the delta time point provides the well DCI:

$$ {\text{DCI}}_{{{\text{ti}}}} = {\text{CI}}_{{{\text{ti}}}} + \left( {{\text{DCI}}_{{{\text{reference}}}} {-}{\text{CI}}_{{\text{delta time}}} } \right) $$

### IncuCyte Cytotox Green and Caspase-3/7 Green

Immortalized human cardiomyocytes were plated (seeding density 2.5 × 10^4^ cells/cm^2^) in 10% FBS-supplemented medium w/o phenol red into a 96 black well plate. The next day the culture medium was replaced with 1% FBS-supplemented medium w/o phenol red, this culture medium was daily replaced until the seventh day of culture. Then, the cells were exposed to 100 and 200 μM of NSAIDs in 1% FBS-supplemented medium w/o phenol red, and 250 nM of IncuCyte Cytotox Green Reagent (Essen BioScience) was added in the experimental culture medium for counting dead cells. For the apoptosis detection, 5 μM IncuCyte Caspase-3/7 Green (Essen BioScience) was added in the experimental culture medium for counting caspases activation The plates were placed in IncuCyte device (20 × objective), the cytotoxicity and caspases activation were recorded (three images for well, six replicates) every 3 h by both phase contrast and fluorescence scanning for 24 h at 37 °C and 5% CO2. Images were analysed using the Incucyte ZOOM software and the data were reported as green object count per mm^2^.

### Western blotting

Crude protein extracts were obtained by RIPA buffer (50 mM Tris pH 8.0, 150 mM NaCl, 0.5% sodium deoxycholate, 0.1% SDS, 1% NP-40 tergitol, 0.12% EDTA and 10 μl/ml of phosphatase inhibitor cocktail 2 and protease inhibitor cocktail, all chemicals purchased from Sigma, St. Louis, Mo, USA) in PBS, after centrifugation at 16,000 *g* for 30 min by refrigerated mini centrifuge, the supernatants were collected and the protein content was assessed by Micro BCA protein detection (Thermo Scientific, Rockford, IL, USA). Treated and untreated cell lysates diluted with 4 × Laemmli sample buffer (BIO-RAD, CA, USA), 30 µg of the total proteins per sample, were run on 4–20% gradient polyacrylamide Mini-PROTEAN TGX Precast Gels (purchased from BIO-RAD, CA, USA) as previously performed by us^78^. The following primary antibodies were used: mouse anti-MyoD (1:500) purchased from Santa Cruz Biotechnology and anti-β-Actin HRP-conjugate 1:10,000 from Cell Signaling Technology, Danvers, MA, USA. After incubation with secondary HRP-conjugate anti-mouse IgG antibody diluted 1:10,000 (Cell Signaling Technology) the immunoreactive bands were visualized by ECL, according to the manufacturer’s instructions (Super Signal West Pico PLUS Chemiluminescent from Thermo Scientific, Rockford, IL, USA). Bands from whole cell lysate obtained using Alliance 4.7 UVITEC (Cambridge, UK) were analysed by ImageJ software and normalized to β-actin, and values were given as relative units (R.U.).

### Measurement of cellular ROS

2′–7′-dichlorofluorescein diacetate (DCFDA) cellular ROS detection assay kit (Abcam, Cambridge, UK) to analyze ROS production in our in vitro model was used according to manufacturer’s instructions. Briefly, immortalized human cardiomyocytes were plated (seeding density 2.5 × 10^4^ cells/cm^2^) in 10% FBS-supplemented medium w/o phenol red into a 96 black well plate. The next day the culture medium was replaced with 1% FBS-supplemented medium w/o phenol red, this culture medium was daily replaced until the seventh day of culture. Then, the cell monolayer was washed one time with 1X buffer, and was incubated with DCFDA 10 μM for 30 min at 37 °C protected from the light. Later the cell monolayer was washed with PBS and the cells were exposed to 100 and 200 μM of NSAIDs in 1% FBS-supplemented medium w/o phenol red. H_2_O_2_ 800 μM was used as a positive control. Every single experiment was performed in quadruplicate. ROS production was immediately determined by measuring the formation of fluorescent dichlorofluorescein (DCF), using a PerkinElmer VICTOR^3^, at an Ex-485 and Em-535 nm. Measurements were done every 30 min for six hours, this being the optimal condition to measure ROS production, as suggested by the manufacturer. The value of fluorescence intensity at each time point is reported. The value reported was obtained by the ratio of fluorescence at a specific time point on fluorescence at time 0, which was measured immediately after DCFDA incubation.

### Determination of mitochondrial membrane potential (MMP)

The mitochondria dye JC-1 (Abcam, USA) was utilized to evaluate the NSAIDs effect on MMP in immortalized human cardiomyocytes, as indicated by the manufacturer. Briefly, cells were plated (seeding density 2.5 × 10^4^ cells/cm^2^) in 10% FBS-supplemented medium w/o phenol red into a 96 black well plate. The next day the culture medium was replaced with 1% FBS-supplemented medium w/o phenol red, this culture medium was daily replaced until the seventh day of culture. Then, the cells were exposed, for 24 h, to 100 and 200 μM of NSAIDs in 1% FBS-supplemented medium w/o phenol red. As depolarization control, FCCP 100 μM for 4 h was used. FCCP acts as an uncoupling agent, thus preventing ATP synthesis. After exposure to treatments, the cell monolayer was washed with PBS and then incubated with JC-1 dye 10 μM for 20 min at 37 °C protected from light. Later, the cell monolayer was rinsed with 1X dilution buffer and the proportionally fluorescence to MMP was immediately measured by using a PerkinElmer VICTOR^3^. Every single experiment was performed in quadruplicate. The fluorescence of the JC-1 aggregate form was measured by setting the Ex-531 and Em-595 nm wavelengths, while the fluorescence of JC-1 monomer form was measured by setting the Ex-485 and Em-535 nm wavelengths. The fluorescence intensity values were expressed as the ratio JC-1 aggregate form/JC-1 monomer form.

### mPTP assay

Proteasome Mitochondrial PT Pore assay kit (Cayman Chemical, USA) to evaluate the permeability of transition pore in inner mitochondrial membrane in immortalized human cardiomyocytes was used according to the manufacturer’s instructions. This kit use a Calcein AM/Cobalt Chloride quenching technique, calcein-AM stain the entire cell, while cobalt chloride is able to quench the calcein fluorescence signal outside the mitochondrial matrix. If the inner mitochondrial membrane (IMM) is in physiological condition cobalt can not cross the IMM and the cells exhibit green fluorescence. Conversely, if the IMM is damaged the green fluorescence from calcein is quenched by cobalt, and the cells exhibit a decrease of green fluorescence intensity. TMRE staining for the measurement of MMP was used, as suggested by the manufacturer. Briefly, immortalized human cardiomyocytes were plated (seeding density 2.5 × 10^4^ cells/cm^2^) in 10% FBS-supplemented medium w/o phenol red into a 96 black well plate. The next day the culture medium was replaced with 1% FBS-supplemented medium w/o phenol red, this culture medium was daily replaced until the seventh day of culture. Then, the cells were exposed, for 12 h (the optimal time to effectively carry out the assay, as suggested by the manufacturer), to 100 and 200 μM of NSAIDs in 1% FBS-supplemented medium w/o phenol red. FCCP 10 μM for 12 h was used as depolarization control, and Calcein Quenching Control 1 μM for 12 h was used as negative control. Then, the plate was placed in IncuCyte device (20 × objective) and the calcein-AM/cobalt chloride (ex/em 485/535) and TMRE (ex/em 545/576) were recorded (three images for well, in six replicates) by both phase contrast and fluorescence scanning at 37 °C and 5% CO2. Images were analyzed using the Incucyte ZOOM software. The specific fluorescence intensity analyzed by Incucyte ZOOM software was reported as Relative Fluorescence Unit (RFU).

### Proteasome (chymotrypsin-like) activity assay

Proteasome activity assay kit (Abcam) to evaluate the chymotrypsin-like activity of the proteasome in immortalized human cardiomyocytes was used according to the manufacturer’s instructions. The chymotrypsin-like activity was determined utilizing an AMC-tagged peptide substrate, which releases free highly fluorescent AMC (7-amido-4-methyl coumarin) in the presence of proteasome proteolytic activity. The assay was performed in the presence and absence of MG132 proteasome inhibitor. Briefly, cells were plated (seeding density 2.5 × 10^4^ cells/cm^2^) in 10% FBS-supplemented medium w/o phenol red into a flask T75 cm^2^. The next day, the culture medium was replaced with 1% FBS-supplemented medium w/o phenol red, this culture medium was daily replaced until the seventh day of culture. On the seventh day of culture, the cells were exposed, for 24 h, to 100 and 200 μM of NSAIDs in 1% FBS-supplemented medium w/o phenol red. After exposure to treatments, the cell monolayer was detached by trypsin and centrifuged 6 min at 250 *g.* The cell pellets were washed with cold PBS and transferred into 1.5 ml tubes, then centrifuged 6 min at 250 *g.* 0.5% NP-40 (tergitol) in PBS was used to suspend cell pellets to obtain protein extract. About 500 μl, for cell pellet, of 0.5% NP-40 extraction buffer was used. After homogenization by pipetting up and down ten times, the extracts were centrifuged 15 min at 16,000 *g* by refrigerated mini centrifuge. The supernatants were collected and maintained at 4 °C, ready for the assay. Extract samples and AMC standards (1–10 μM) were placed in 96 black well plate in a total volume of 100 μl. In all sample wells, the fluorescent substrate AMC (final concentration 50 μM) was placed with or without MG132 proteasome inhibitor (final concentration 100 μM). After mixing all the components in the wells, the plate was incubated at 37 °C for 20 min protected from light (T1 measure). Chymotrypsin-like activity at T1 was determined by measuring the fluorescence released from the AMC substrates, using a PerkinElmer VICTOR^3^, at an Ex-355 and Em-460 nm. After the first measurement, the plate was incubated at 37 °C for 30 min protected from light (T2 measure). Chymotrypsin-like activity at T2 was determined by measuring the fluorescence released from the AMC substrates, using a PerkinElmer VICTOR^3^, at an Ex-355 and Em-460 nm. Jurkat cell lysate, with significant proteasome activity, was used as a positive control, and each experiment was performed in triplicate. To quantify proteasome activity, described as “one unit of proteasome activity is defined as the amount of proteasome which generates 1 nmol of AMC per minute at 37 °C”, the manufacturer’s instructions were followed. First, at each T (T1 or T2), the fluorescence values from the wells without inhibitor were subtracted to the fluorescence values from the wells with inhibitor, to obtain tRFU (total relative fluorescence unit). Measurement of the well without the proteasome inhibitor showed total proteolytic activity, and the wells containing proteasome inhibitor showed non-proteasome activity. Then, delta RFU = tRFU2–tRFU1 was calculated. Delta RFU values were applied to the AMC standard curve to obtain B, which is the amount of AMC in the sample well expressed as pmol/well. Proteasome activity was obtained by:

$$ {\text{Proteasome activity}} = \left( {{\text{B}}/\left( {{\text{T}}2 - {\text{T}}1} \right)*V} \right)*D, $$

where B is the amount of AMC (pmol) in the sample, calculated by the AMC standard curve. V is the total volume reaction (μl) in the well; T1 and T2 are the time (min) of the first and second readings, respectively. D is the sample dilution factor.

### Proteasome (trypsin-like and caspase-like) activity assay

Proteasome activity assay to evaluate trypsin-like and caspase-like activity of the proteasome in immortalized human cardiomyocytes was used. Trypsin-like and caspase-like activities were determined utilizing an AMC-tagged peptide substrate, which releases free highly fluorescent AMC (7-amido-4-methyl coumarin) in the presence of proteasome proteolytic activity. The assay was performed in the presence and absence of bortezomib (Santa Cruz Biotechnology, Dallas, TX, USA) proteasome inhibitor. Briefly, cells were plated (seeding density 2.5 × 10^4^ cells/cm^2^) in 10% FBS-supplemented medium w/o phenol red into a flask T75 cm^2^. The next day, the culture medium was replaced with 1% FBS-supplemented medium w/o phenol red, this culture medium was daily replaced until the seventh day of culture. On the seventh day of culture, the cells were exposed, for 24 h, to 100 and 200 μM of NSAIDs in 1% FBS-supplemented medium w/o phenol red. After exposure to treatments, the cell monolayer was detached by trypsin and centrifuged 6 min at 250 *g*. The cell pellets were washed with cold PBS and transferred into 1.5 ml tubes, then centrifuged 6 min at 250 *g.* Proteasome lysis buffer (50 mM Tris–Hcl pH 7.5, 250 mM sucrose, 5 mM MgCl_2_, 0.5 mM EDTA free acid, 1 mM DTT, 2 mM ATP, 0.025% digitonin, 10% glycerol, all chemicals were purchased from all chemicals purchased from Sigma, St. Louis, Mo, USA) in Milli-Q-water was used to suspend cell pellets, to obtain crude protein extract. About 120 μl, for cell pellets, of proteasome lysis buffer were used. After homogenization by pipetting up and down ten times, the extracts were incubated 10 min at 4 °C. After centrifugation 30 min at 20,000 *g* (by refrigerated mini centrifuge), the supernatants (protein crude extract) were collected and maintained at 4 °C, ready for the assay. The total protein content was determined by extrapolation from a BSA standard curve (0.025–2 mg/ml). Protein crude extracts were dilute in proteasome assay buffer (50 mM Tris–HCl pH 7.5, 40 mM KCl, 5 mM MgCl_2_, 0.5 mM ATP, 1 mM DTT, 0.05 mg/ml BSA in Milli-Q-water) to obtain 5–10 μg/100 μl of final protein concentration into the well. Extract samples and AMC standards (1–10 μM) were placed in 96 black well plate in a total volume of 100 μl. In all sample wells the fluorescent substrate AMC (final concentration 200 μM, trypsin-like [Boc-LRR-AMC] and caspase-like [Z-LLE-AMC] both from R&D System, Minneapolis, MN, USA) was placed with or without bortezomib proteasome inhibitor (final concentration 100 μM). After mixing all the components in the wells, the plate was incubated at 37 °C for 30 min protected from light (T1 measure). Trypsin-like and caspase-like activity at T1 was determined by measuring the fluorescence released from the AMC substrates, using a PerkinElmer VICTOR^3^, at an Ex-355 and Em-460 nm. After the first measurement, the plate was incubated at 37 °C for other 30 min protected from light (T2 measure). Trypsin-like and caspase-like activity at T2 was determined by measuring the fluorescence released from the AMC substrates, using a PerkinElmer VICTOR^3^, at an Ex-355 and Em-460 nm, each experiment was performed in triplicate. To quantify specific proteasome activity at each T (T1 or T2), the fluorescence values from the wells without inhibitor were subtracted to the fluorescence values from the wells with inhibitor, to obtain total relative fluorescence unit (tRFU). Measurement of the well without proteasome inhibitor showed total proteolytic activity and the wells containing proteasome inhibitor showed non-proteasome activity. Then delta RFU = tRFU2–tRFU1 was calculated. Delta RFU values were applied to the AMC standard curve to obtain B, which is the amount of AMC in the sample well, expressed as pmol/well. Proteasome activity was obtained by:

$$ {\text{Proteasome activity}} = \left( {{\text{B}}/\left( {{\text{T2}} - {\text{T1}}} \right)*{\text{V}}} \right), $$

where B is the amount of AMC (pmol) in the sample, calculated by the AMC standard curve. V is the total volume reaction (μl) in the well, T1 and T2 are the time (min) of the first and second reading, respectively.

### Characterization of proteasomes

Characterization of different proteasome structures was done by native gel electrophoresis/western blotting (5%), as already described^33,38^. Briefly, immortalized human cardiomyocytes were plated (seeding density 2.5 × 10^4^ cells/cm^2^) in 10% FBS-supplemented medium w/o phenol red into a flask T75 cm^2^. The next day, the culture medium was replaced with 1% FBS-supplemented medium w/o phenol red, this culture medium was daily replaced until the seventh day of culture. The cells were exposed, for 24 h, to 100 and 200 μM of NSAIDs in 1% FBS-supplemented medium w/o phenol red. Later, the cell pellets were collected and homogenized in lysis buffer containing: 50 mM Tris–HCL pH 7.5, 5 mM MgCl_2_, 0.5 mM EDTA, 2 mM ATP and 0.5% NP-40 (tergitol) in Milli-Q-water. For cell homogenization, pellets were vortexed for 5 min at 4 °C and then incubated for 30 min at 4 °C. After centrifugation at 15,000 *g* and 4 °C for 30 min the supernatants were collected and stored at − 20 °C. The total protein content was determined by extrapolation from a BSA standard curve (0.025–2 mg/ml). Later, the samples were diluted 1:1 in native sample buffer (Bio-Rad) and then were loaded in native gel condition. The run gel was composed of stacking upper gel (3.5%) and resolving gel (5%) with freshly added 1 mM ATP. Electrophoresis was carried out in TBE buffer (90 mM Tris, 90 mM Borate, 0.5 mM EDTA and freshly added MgCl_2_ 5 mM and 0.5 mM ATP, all chemicals were purchased from Sigma, St. Louis, Mo, USA) at 50 V for 40 min, 100 V for 30 min and 150 V for 3 h. Blotting buffer 25 mM Tris, 192 mM Glycine, and 20% Methanol (Sigma, St. Louis, Mo, USA) was used to transfer the proteins onto PVDF membranes (Thermo Scientific, Rockford, IL, USA) through wet electrophoretic transfer (constant 400 mA for 2 h and 30 min). After blocking non-specific by 5% (w/v) non-fat dry milk in TBS-T 0.1% (Santa Cruz Biotechnology, Dallas, TX, USA) the membranes were incubated with rabbit anti-human PSMA6 (1:5000) (Abcam). Later, incubation with secondary HRP-conjugated anti-rabbit IgG antibody diluted 1:10,000 (Cell Signaling Technology, Danvers, MA, USA) was performed and the immunoreactive bands were visualized by ECL, according to the manufacturer’s instructions. Bands from whole cell lysate obtained using Alliance 4.7 UVITEC (Cambridge, UK) were analyzed by ImageJ software, and values were given as relative proportion %. Briefly, data were expressed as % of the ratio between PSMA6 20S and PSMA6 26SDC, respectively, on tPSMA6 (total PSMA6 = PSMA6 20S + PSMA6 26SDC).

### OxyBlot assay

OxyBlot protein oxidation detection kit (Merck Millipore, Burlington, MA, USA) to evaluate oxidized protein levels in immortalized human cardiomyocytes were used according to the manufacturer’s instructions. Briefly, cells were plated (seeding density 2.5 × 10^4^ cells/cm^2^) in 10% FBS-supplemented medium w/o phenol red into a flask T75 cm^2^. Later, the culture medium was replaced with 1% FBS-supplemented medium w/o phenol red, this culture medium was daily replaced until the seventh day of culture. On the seventh day of culture, the cells were exposed, for 24 h, to 100 and 200 μM of NSAIDs in 1% FBS-supplemented medium w/o phenol red. Subsequently, the cell pellets were collected and homogenized in lysis buffer (provided in the kit) containing DTT 50 mM. After protein extraction, 5 μg/μl of protein extracts were used to perform derivatization, as suggested by the manufacturer. Negative control (Neg. Ctr) is a sample without derivatization. Lysates from control and treated cells (20 µg total proteins per sample) were run on 10% polyacrylamide SDS denaturing gels, as previously performed by us^78^. The following primary antibody was used: rabbit anti-DNP diluted 1:150. After incubation with secondary HRP-conjugated anti-rabbit IgG antibody diluted 1:300 the immunoreactive bands were visualized by ECL, according to the manufacturer’s instructions. Bands from whole cell lysate obtained using Alliance 4.7 UVITEC (Cambridge, UK) were analyzed by ImageJ software and normalized to blue Coomassie staining and values were given as relative units (R.U.).

### Statistical analyses

Data are expressed as mean ± standard error mean (SEM). Samples were processed by Graph Pad Prism 6 software (RRID: SCR_002798). Two-tailed unpaired student’s t-test Welch-corrected was used to determine statistical differences among groups. A *p* value of < 0.05 was considered statistically significant.

## Supplementary information

Supplementary Figures.

## Abbreviations

NSAIDs: Non-steroidal anti-inflammatory drugs
OTC: Over-the-counter
COXs: Cyclooxygenase
PGs: Prostaglandins
PGI2s: Prostacyclins
TXs: Thromboxanes
tNSAIDs: Traditional non-steroidal anti-inflammatory drugs
AMI: Acute myocardial infarction
HF: Heart failure
IS: Acute ischemic stroke
ROS: Reactive oxygen species
MMP: Mitochondrial membrane potential
mPTPs: Mitochondrial permeability transition pores
K: Ketoprofen
Dic: Diclofenac
MHC7: Mouse anti-myosin heavy chain 7
Cx43: Connexin 43
MyoD: Myoblast determination protein 1
CI: Cell index
DCI: Delta cell index
UPS: Ubiquitin–proteasome system
CP: Core particle
RP: Regulatory particle
PSMA6: Proteasome sub-unit α6
26S DC: Proteasome 26S double capped
EP: E-type prostanoids
20-HETE: 20-Hydroxyeicosatetraenoic acid
