# Supplementary material for: NSAIDs-dependent adaption of the mitochondria-proteasome system in immortalized human cardiomyocytes
Source: Sci Rep. 2020 Oct 27;10:18337. doi: 10.1038/s41598-020-75394-x (PMC7591859; doi:10.1038/s41598-020-75394-x)
Supplement: Supplementary file 1 — Supplementary Figures. [file 41598_2020_75394_MOESM1_ESM.docx]

**NSAIDs-dependent adaption of the mitochondria-proteasome system in immortalized human cardiomyocytes**

Laura Brandolini^1^, Andrea Antonosante^2^, Cristina Giorgio^1^, Michela Bagnasco^1^, Michele d’Angelo^2^, Vanessa Castelli^2^, Elisabetta Benedetti^2^, Annamaria Cimini^2,3^, Marcello Allegretti^1^.

^1^ Dompé Farmaceutici SpA, Via Campo di Pile, L’Aquila, Italy

^2^ Department of Life, Health and Environmental Sciences, University of L’Aquila, Italy

^3^ Sbarro Institute for Cancer Research and Molecular Medicine and Centre for Biotechnology, Temple University, Philadelphia, USA

**Supplementary information**

**Supplementary Figure S1**


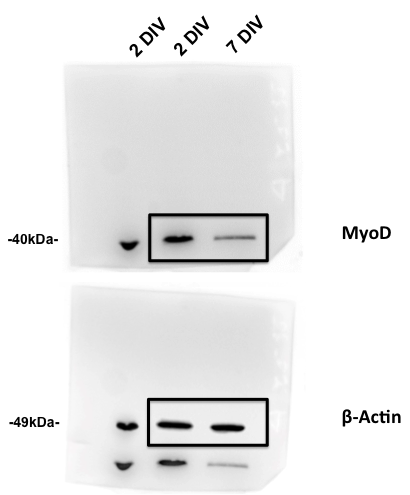


Supplementary figure S1. Full length Western blots. The boxed regions were presented in Fig. 1D. In our experimental conditions, Myod appeared about at 40 kDa, while the Actin (housekeeping, incubated the day after) is about 49 kDa. We loaded twice 2 DIV conditions (1^st^ and 2^nd^ lanes), the third sample represents 7 DIV. In the representative figure we chose to show 2^nd^ and 3^rd^ lanes. Altough in our experimental conditions MyoD appeared about at 40 kDa, in the figure into the paper we preferred to indicate 45kDa, which is the molecular weight referred to the data sheet provided from the company (Santa Cruz Biothecnology, MyoD (G-1): sc-377460).

**Supplementary Figure S2**

Supplementary figure S2. Full length Western blots of native-gel electrophoresis presented in Fig. 7A, B. We cannot indicate the specific molecular weight of the band, it is due to the very high molecular weight of the structures recognized by anti-PSMA6 antibody. Molecular mass of these proteasome structures is about 2500 kDa for proteasome 26DC and 750 kDa for proteasome 20S.

**Supplementary Figure S3**


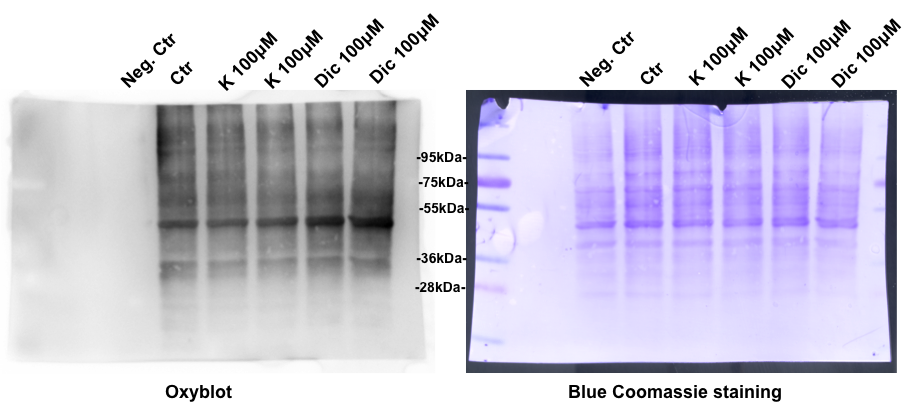


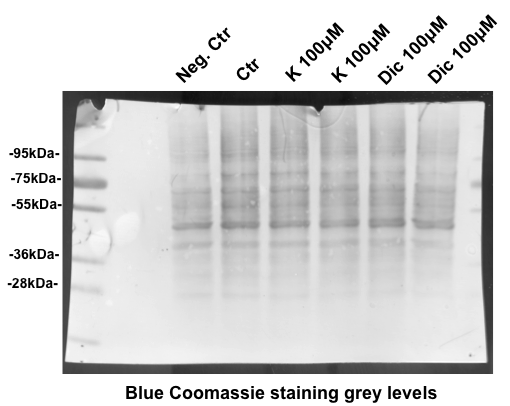


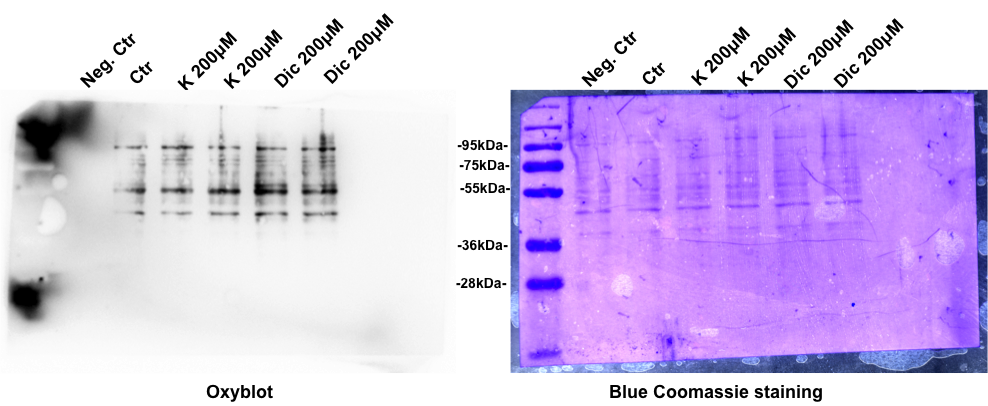


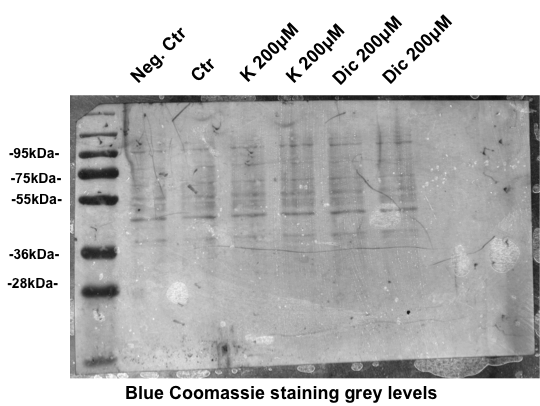


Supplementary figure S3. Full length Western blots and membrane stained with Blue coomassie presented in Fig. 8A-B.
